# Editorial: New therapeutics for soft tissue sarcomas

**DOI:** 10.3389/fonc.2023.1297215

**Published:** 2023-10-16

**Authors:** Marla Weetall, Mark Rance, Matthew Ingham, Brian A. Van Tine

**Affiliations:** ^1^ Pharmacology and Biomarkers, PTC Therapeutics (United States), South Plainfield, NJ, United States; ^2^ Clinical Development, Regeneron Pharmaceuticals, Inc., Tarrytown, NY, United States; ^3^ Division of Medical Oncology, Washington University in St. Louis, St. Louis, MO, United States; ^4^ Siteman Cancer Center, St. Louis, MO, United States; ^5^ Division of Pediatric Hematology and Oncology, St. Louis Children's Hospital, St. Louis, MO, United States

**Keywords:** sarcoma, combination (combined) therapy, chemotherapy, immunotherapy, personalized medicine

Soft tissue sarcomas (STS) represent less than 1% of all tumors and are thought to be derived from mesenchymal progenitor cells. This heterogeneous class of tumors include over 100 histological subtypes with varying clinical presentation and genetics. Their heterogeneity reflects the ability of mesenchymal precursors to develop into an array of tissue types including muscle, bone, cartilage, and fat.

There are no routine standard screening tests for sarcoma, but once a tumor is detected the prognosis is based on the disease stage, histopathology, size, and genetics. Standard of care includes surgery, radiation and chemotherapy. A percentage of sarcomas have actionable driver mutations, but most do not. STS are typically “cold”, with a low tumor burden and are not likely to respond to immunotherapy. Despite improvements in therapy, the 5-year survival rate remains low, reported as 58% (Cancer Net, ASCO) due in part to higher frequencies of advanced metastatic disease.

This Research Topic includes nine multidisciplinary manuscripts. One overall theme is that targeted therapies can be very effective for specific STS with specific actionable mutations, but only a limited percentage of sarcoma patients have these mutations. Even patients that respond well to targeted therapy often relapse, and then require subsequent non-targeted therapy. Therefore, additional therapies that are both for targeted sarcomas and for sarcomas lacking obvious actionable genetics are required that can prevent relapse/prolong PFS.

Fuchs et al., reviewed FDA approved drugs for STS. Pazopanib is FDA approved for non-adipocyte STS based on clinical prolongation of the PFS from 1.6 months to 4.6 months. The OS increase did not reach statistical significance (10.7 vs 12.5 months). Other drugs reviewed were approved for more targeted populations and have longer response rates. (1) Pexidartinib (CSF1R, Ckit inhibitor) for tenosynovial giant-cell tumors (locally invasive, frequently due to CSF1 overexpression), 39% overall response rate (2) imatinib for dermatofibrosarcoma protuberans (locally invasive with COL1A1/PDGFB fusion protein), 5% CR, 55% PR, (3) crizotinib for ALK positive inflammatory myofibroblastic tumors (locally invasive), (4) tazemetostat (EZH2 inhibitor) for epithelioid sarcoma (can be metastatic; characterized by loss of INI1 subunit of the SWI/SNF chromatin remodeling complex that opposes the enzymatic function of EZH2), (5) nab-sirolimus for perivascular epithelioid cell tumor (PEComa, often seen with tuberous sclerosis), (6) tropomyosin receptor kinase inhibitors for TRK fusion positive cancers. Also reviewed were targeted kinase inhibitors that do not have FDA approval for STS but are in clinical testing.

Maleddu et al. reviewed only locally aggressive mesenchymal tumors, particularly Desmoid fibromatosis, giant cell tumor of bone, and tenosynovial giant cell tumor. These tumors have limited ability to metastasize and therefore carry a better prognosis than metastatic tumors. As noted by Fuchs et al. as well, treatment for these includes targeted chemotherapy approaches that can be effective. New therapies and paradigms have focused on utilizing less toxic regimens and identifying patients that do not require more toxic regimens.

Fuchs et al. reviewed new therapeutics for synovial sarcoma (SYN). SYN is defined by the translocation of t(X:18) (p11.2;q11.2) forming an oncogenic fusion protein, with about 1000 cases per year in the US (1). Although SYN is more responsive to chemotherapy, the overall survival is worse than that of most STS. He reviewed clinical trials using adoptive cell transfer, where autologous T cells are transfected with engineered T-cell receptors that bind antigens that are expressed predominately in SYN including NY-ESO-1, PRAME, and MAGE-A4. Included is a description of the challenges with the approach.

Seong and D’Angelo reviewed immune approaches for STS. STS generally have low levels of infiltrating lymphocytes and may have higher levels of immunosuppressive M2 macrophages.

Lacuna et al. reviewed new therapeutics for leiomyosarcoma (LMS). First-line therapies are anthracycline- or gemcitabine-based regimens, resulting in a median PFS time of about 5 months and overall survival time between 14-16 months. LMS is not typically associated with specific mutations, but with complex karyotypes. Highlighted in the review are new therapies based upon: (1) DNA repair deficiencies because LMS is often associated with defects in DNA-repair. This includes a Ph2/3 trial assessing temozolomide and Olaparib in uterine LMS. (2) Kinase inhibitors, including cabozantinib and anlotinib. (3) Metabolic vulnerabilities due to the frequent loss of argininosuccinate synthase 1. (4) New chemotherapeutic combinations including a Phase 3 trial of unesbulin with dazacarbazine. Unesbulin is an orally bioavailable inhibitor of tubulin polymerization. Possibly, the combination of unesbulin (causes G2M arrest) and dazacarbazine (an alkylating agent) causes replicative stress, particularly in tumors such as LMS that are deficient in DNA-repair.

Three papers discuss new therapies tested in preclinical studies or in a case report. First, Bernardo et al. demonstrates that both photon and proton irradiation are equally effective in a sarcoma mouse model. In clinical trials, first line therapy is the combination of radiotherapy with doxorubicin, although the rates of recurrence remain high. Clinical trials are not conducted to optimize radiation/chemotherapy regimens or to compare types of radiation, increasing their value in preclinical studies. Second, Marritt et al. present preclinical data demonstrating efficacy of STING agonists in models of undifferentiated polymorphic sarcoma UPS. Third, Li et al. describe a case report of a favorable response of an ALK-fusion positive protein to the ALK TKI ensartinib. Epithelioid inflammatory myofibroblastic sarcoma (EIMS) is often associated with ALK fusion proteins.

Finally, because osteosarcomas show genetic similarities to soft tissue sarcomas, we have included a paper describing a prognostic score predicting responsiveness to high dose methotrexate (Ganguly et al.). Tumor size, baseline metastases and SAP were prognostic factors to predict survival, but social factors were not. Similar parameters are likely to be relevant to STS tumors.

In summary, new therapies are being identified and developed for sarcomas. Some of these are targeted therapies leveraging the gains made in personalized medicine. Others are focused on novel chemotherapeutic combinations leveraging the complex karyotype of sarcoma on the background of DNA repair deficiencies. Exciting new immuno-oncology approaches are in preclinical and clinical stages. Some of these approaches may provide new therapies to overcome resistance and provide more possible treatments to mix and match in varying sequence so as to provide measurable benefit to STS patients.

## Author contributions

MW: Conceptualization, Writing – original draft, Writing – review & editing. MR: Writing – original draft, Writing – review & editing. MI: Conceptualization, Writing – review & editing. BT: Conceptualization, Writing – review & editing.
